# Editor’s Note: Domain-general cognitive motivation: evidence from economic decision-making

**DOI:** 10.1186/s41235-021-00308-y

**Published:** 2021-06-04

**Editor’s Note**

This article is an inadvertent early publication of a pre-registered report. After the pre-registration was accepted, but before the experimental work was completed, the article was accidentally published in the journal. The full article will be forthcoming and will be linked to this article [1].
